# 

**Published:** 1888-12

**Authors:** 


### CONTENTS.

### COMMUNICATIONS.

The Ottoleungui Method of Anæsthetizing Sensitive Dentine.......... 577
Pericementitis........................................................ 587
Medical Education for Dentists...................................... 597
Disintegration at the Cervical Borders.......................... 608
Anæsthetics in Dental Practice...................................... 613
Continuous Gum Dentures............................................ 616

### SELECTIONS.

Sensitive Dentine........................................................ 618
The Air of the Bed Room.............................................. 620
Skin Grafting.............................................................. 622
Tobacco on the Eyes.................................................... 622
Danger in Large Doses of Quinine................................. 623
Six Children at a Single Birth....................................... 623

### EDITORIAL.

College Libraries......................................................... 623
Crown and Bridge Work................................................ 624

